# Multicompartmental Lipopolyplex as Vehicle for Antigens and Genes Delivery in Vaccine Formulations

**DOI:** 10.3390/pharmaceutics13020281

**Published:** 2021-02-19

**Authors:** Isaías Sanmartín, Luis Sendra, Inés Moret, María José Herrero, Salvador F. Aliño

**Affiliations:** 1 Faculty of Veterinary and Experimental Sciences, Universidad Católica de Valencia, 46001 Valencia, Spain; isaias.sanmartin@ucv.es; 2 Pharmacology Department, Faculty of Medicine, Universidad de Valencia, 46010 Valencia, Spain; luis.sendra@uv.es (L.S.); ines.moret@uv.es (I.M.); maria.jose.herrero@uv.es (M.J.H.); 3 Pharmacogenetics Unit, Instituto de Investigación Sanitaria La Fe, 46026 Valencia, Spain; 4 Inflammatory Bowel Disease Research Group, Instituto de Investigación Sanitaria La Fe, 46026 Valencia, Spain; 5 Clinical Pharmacology Unit, Hospital Universitario y Politécnico La Fe, 46026 Valencia, Spain

**Keywords:** lipopolyplexes, multicompartmental lipopolyplexes, melanoma, non-viral gene transfer, antitumor immunization, vaccine, liposomes

## Abstract

Vector design and its characterization is an area of great interest in current vaccine research. In this article, we have formulated and characterized a multicompartmental lipopolyplex, which associates multiple liposomes and polyplexes in the same complex. These particles allow the simultaneous delivery of lipid or water-soluble antigens associated with genes to the same cell, in much higher amounts than conventional lipopolyplexes. The vector characterization and optimization were carried out using liposomes with entrapped carboxyfluorescein and adapted electrophoretic assays. Two types of lipopolyplexes (containing hydrophilic or lipophilic antigens) were employed to evaluate their interest in vaccination. The lipopolyplex loaded with an extract of water-soluble melanoma proteins proved to efficiently induce humoral response in murine melanoma model, increasing the levels of IgM and IgG. The specificity of the immune response induced by the lipopolyplex was demonstrated in mice with the lipopolyplex containing the GD3 ganglioside lipid antigen, abundant in melanoma cells. The levels of anti-GD3 IgG increased markedly without modifying the expression of humoral antibodies against other gangliosides.

## 1. Introduction

Liposomes and polycationic polymers are the most used systems as non-viral gene transfer agents. Lipopolyplexes (LPP) represent an evolution of the previous ones and they have experienced a boom as gene transfer vehicles in recent years, particularly for their use as immunizing agents. These lipopolyplexes associate lipids, cationic polymers, and nucleic acids, offering better transfection properties and lower cytotoxicity [1,2]. The final structure of LPPs depends on the employed components, the polymer and liposome electronic charge, and the molecules to be carried. All these elements, and the order in which they are added, must play important roles in the LPP constitution. We consider that the independent location of carried elements in different compartments should offer an important improvement for LPP stability and delivery control. The structure of a lipopolyplex is similar to the organization of an enveloped viral particle: A DNA or RNA molecule is compacted by its interaction with a polycation polymer into a nano-sized particle conforming the ‘core’ of the lipopolyplex. This is located inside a lipid vesicle so that it is surrounded by a lipid bilayer in a structure similar to that of a liposome vesicle.

Most of the vehicles called “lipopolyplexes” reported in the current literature have this basic structure [3,4,5,6]. This design allows the generation of particles that present similarities with a viral particle, especially their small size and organization. This is a fundamental aspect in the design of vectors for gene transfer, in which the vehicles need to be small enough to cross the capillary barrier to reach their target cells and cross the cell membrane [7]. Compared to soluble drugs administered parenterally, targeted liposomes have the advantage as a drug delivery vehicle that they can deliver a higher amount of molecules to target cells than those that could reach the cell by diffusion in the extracellular fluid after parenteral administration. This is important because it permits lowering the administered doses required, reducing the potential secondary and side effects of the drug [7,8,9]. These lipopolyplexes have a liposome-like structure but a polycation-complexed DNA molecule occupies the internal core. Therefore, these lipopolyplexes present low potential capacity for antigen transport and this limits their use in immunization. Unlike liposomes, the manufacturing technology of these lipopolyplexes does not allow their direct load with antigens because they are limited to transport the polyplex core, which occupies most of the available space inside the vesicle. However, the size of lipopolyplexes used for immunization does not have to be restricted to nanometers since these do not need to diffuse and distribute throughout the body. A larger immunization vehicle, in the order of microns, can be applied subcutaneously and be phagocytosed by the immune defense cells, such as macrophages and dendritic cells.

**Figure 1:**
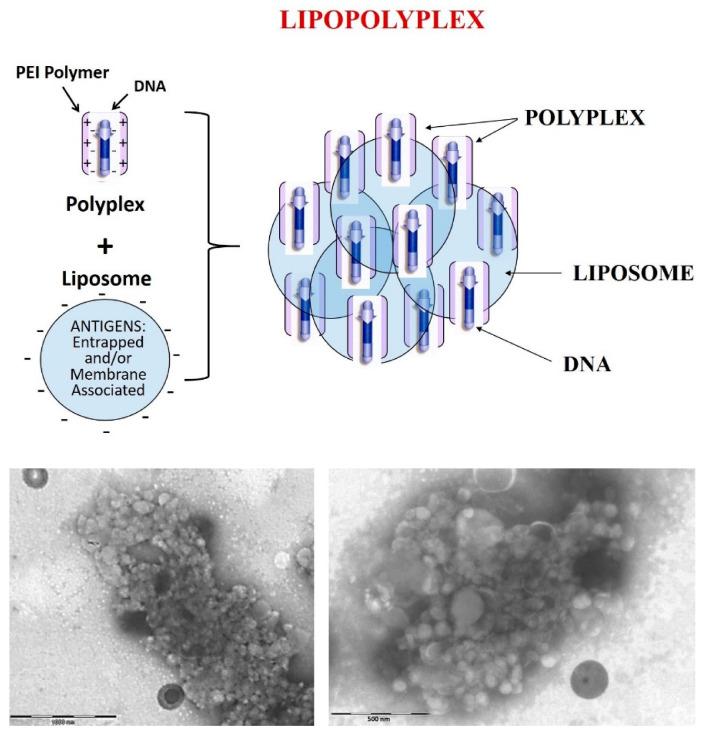
Lipopolyplex structure. Upper: Theoretical diagram of lipopolyplex: Lower panel: TEM images of LPP. Lipopolyplexes images of transmission electron microscopy. Lipopolyplex samples were stained with phosphotungstic acid, which causes negative liposome staining. Complexes size is approximately 1–5 µm in diameter. Left panel scale bar: 1000 nm; right panel scale bar: 500 nm.

In this study, we present a lipopolyplex with multicompartmental structure. In the upper panel of Figure 1, we suggest a theoretical diagram of this complex. In the lower part of Figure 1, the TEM images show the lipopolyplex structure, which contain multiple liposomes (negatively stained) surrounding more electro-dense areas where the polyplex (DNA-PEI) should be located. The independence of each compartment was confirmed by an electrophoretic procedure developed in our laboratory. This technique combines a previously described electrophoresis analysis with heparin-treated polyplexes [10] but adapted to combine the evaluation of size, fluorescence, and quenching capacity. Unlike others that consist in a single vesicle surrounding a polycation core, our vehicle associates multiple lipid vesicles (containing antigens) and polyplexes (nucleic acid and polycation molecules), forming a complex with high capacity for carrying antigens and genes molecules. Each of the multicompartmental lipopolyplex particles can contain numerous polyplexes and liposomes. Therefore, water-soluble antigenic proteins can be entrapped inside the lipid vesicles and lipid-soluble antigens can be located in the lipid bilayer of the vesicle. The lipopolyplexe structure and size was observed by transmission electron microscopy. The multiple polyplexes that associate with liposomes contain numerous plasmid molecules. All these elements are simultaneously incorporated into the same cell that uptakes the multicompartmental lipopolyplex particle and this constitutes a substantial improvement in their carrying capacity with respect to the individual lipopolyplex. Our design allows for the simultaneous transport of large quantities of both nucleic acids and antigens (water- and/or lipid-soluble) to the immune system cells, which is an advantage for their use in vaccination.

As a proof of concept, we have tested our vehicle in a murine melanoma model, with water-soluble antigens derived from tumor membrane proteins (TMP) of murine melanoma cells, or lipid-soluble antigen (the ganglioside GD3), as a component of liposome formulation. For the polyplex component, we employed polyethyleneimine (PEI, 25 KDa) and a DNA plasmid containing the gene encoding for the murine immunostimulating cytokine GM-CSF (Granulocytes and Macrophages Colony Stimulating Factor), as an adjuvant. The GM-CSF gene is interesting in DNA vaccines due to the ability of the cytokine to stimulate dendritic cells (DC) maturation and antigen-presenting cells (APC) recruitment to the site of administration. The GM-CSF is largely demonstrated as a strong adjuvant in antitumor immunization assays [11,12,13,14,15,16].

To test the capacity of our vehicle to transport water-soluble antigens, the lipid vesicles were loaded with TMP, an extract of water-soluble proteins obtained from melanoma cells. In previous works of our laboratory, sphingomyelin or phosphatidylcholine liposomes encapsulating a TMP extract from B16 cells had shown to reduce significantly the number of lung metastases in immunization experiments with C57BL/6 mice [17]. A multicompartmental lipopolyplex was prepared with these vesicles and assayed in a multiple antigen vaccination trial.

To evaluate the capacity of our vehicle to transport lipid-soluble antigens and mediate antigen specific immune response, the lipid vesicles were prepared with the GD3 ganglioside. This lipopolysaccharide is present in abnormally high quantities in melanoma cells and has proved to be able to generate antigenic response [18,19]. We located it within the lipid bilayer of the liposomal component of the multicompartmental lipopolyplex, which was employed in an antigen vaccination trial in mice.

Although no significant effects on tumor growth and survival rate were observed, both the vaccine with the TMP hydrophilic antigen and the vaccine with the GD3 lipophilic antigen have been shown to activate highly significantly the humoral immune response, augmenting the production of total IgG and especially IgG2a.

## 2. Materials and Methods

### 2.1. Materials

#### 2.1.1. Reagents

Branched polyethylenimine (PEI) 25 kDa, Sepharose 4B, 5(6) carboxyfluorescein mixed isomers (CF), sphyngomyelin (SM), dicetylphosphate (DP), and cholesterol (CH) were purchased from Sigma-Aldrich (Madrid, Spain). Ganglioside GD3, Disialo, and Diammonium Salt were from Calbiochem^®^ (Barcelona, Spain). Agarose D-1 media EEO was from Hispanlab, S.A (Madrid, Spain).

#### 2.1.2. Plasmids

pMOK-GMCSF (containing GM-CSF mouse gene controlled by CMV promoter) and P2F and p2F m-GM-CSF (containing the murine GM-CSF protein cDNA driven by ferritin promoter) were both purchased at Invitrogen (Madrid, Spain). Plasmids were amplified by cloning them in DH5a strain of E. coli and purified by using Qiagen EndoFree Giga-Prep kits (Qiagen, Valencia, CA, USA) following the manufacturer instructions. The plasmids were checked by agarose electrophoresis and spectrophotometric A260/A280 ratio to assess purity and integrity before use.

#### 2.1.3. Animals

Female C57BL/6 mice (7–10 weeks old) were obtained from Harlan (Gannat, France). The animals were housed in groups of four to five per cage under standard laboratory conditions. The experimental project was approved by the Biological Research Committee of the University of Valencia (Valencia, Spain), with reference number A1319118959093.

### 2.2. Methods

#### 2.2.1. Proteins Extraction and Quantitation

Proteins used as antigens in immunization experiments were obtained according to the Bordier extraction protocol [20] from murine B16 melanoma cells. B16 cells were resuspended in a ratio of 1 mL buffer/1,000,000 cells in “extraction buffer” (10 mM Tris, 2 mM MgCl_2_, 0.5% Triton X-114, 0.1 mM PMSF, pH 7.2), incubated on ice for 45 min (shaking the mixture gently every 10 min), and centrifuged for 15 min at 5300 rpm and 4 °C. The supernatant (750 mL) was mixed with 250 mL of “centrifugation buffer” (10% sucrose in extraction buffer) and incubated for 3 min at 37 °C. The tubes were then centrifuged for 5 min at 2100 rpm and 750 mL of the new supernatant were separated. Triton X-114 was added to a final concentration of 0.5% and then mixed with 250 mL of ice-cooled centrifugation buffer. The tubes were incubated for 3 min at 37 °C and centrifuged again for 5 min at 2100 rpm, obtaining two phases: The upper one contains the proteins, which are separated and kept at −20 °C until use.

#### 2.2.2. Preparation of Polyplexes

The PEI/DNA polyplex was prepared in 5% glucose solution at 10/1 (+/−) charge ratio, which corresponds to 1 mg DNA/1.4 mg PEI mass ratio. A 7 mg/mL stock solution of PEI 25KDa (Sigma Aldrich Cat. no. 408727, St. Louis, MO, United States) was prepared by gravimetry dissolving the PEI in distilled water in a beaker on a balance. The pH was adjusted to 7.6 by HCl addition. From this stock, a working solution of 1.4 mg/mL PEI was made in 5% glucose. Plasmid DNA was dissolved to 1 mg/mL in 5% glucose. Equal volumes of plasmid and PEI solutions were mixed and immediately shaken with the vortex and incubated for 15 min before liposome addition. Final DNA concentration was 0.5 µg/µL in the polyplex preparation.

#### 2.2.3. Preparation of Liposomes

The lipid mixture in appropriate molar ratios was dissolved in chloroform and evaporated in a round bottom flask under nitrogen flux, forming a dry lipid film on the flask bottom. In a typical liposomal preparation, a solution of 50 µmoles of lipids in 300 µL of chloroform was evaporated forming the dry lipid film, and the last traces of organic solvent were removed under vacuum during 60 min.

We prepared MLV and MLV-FT anionic liposomes of different composition (PC:CH:DP 5:4:1 and SM:CH:DP 5:4:1) for vaccines with hydrosoluble antigens (TMP) and MLV and SUV anionic liposomes (SM:CH:GD3 5:4:1 and SM:CH:DP:GD3 4:4:1:1) for vaccines with lipid antigens. For the electrophoretic assays with carboxyfluorescein fluorescent liposomes, SUV liposomes of composition SM:CH:DP 5:4:1 were prepared.

For multilamellar lipidic vesicles preparation (MLV), the dry lipid film was redispersed in 500 µL of the aqueous solvent and vigorously shaken with the vortex until complete lipid redispersion. For small unilamellar vesicles preparation (SUV), a previously prepared MLV liposome suspension was sonicated to afford small unilamellar, as evidenced by the clarification of the suspension. Thereafter, titanium particles released from the probe and residual MLV liposomes were removed by centrifugation (20,000 g × 20 min). For freeze-thawed multilamellar lipidic vesicles preparation (MLV-FT), a previous MLV encapsulating TMP liposome suspension was frozen in liquid nitrogen (−196 °C) and then thawed in a thermostated bath at 37 °C. This process was repeated 5 times to obtain the MLV-FT liposomes. In the case of MLV-FT liposomes encapsulating TMP, free non-encapsulated proteins were removed by centrifugation (20,000 g × 20 min).

For the carboxyfluorescein fluorescent liposomes, dry lipid film was re-dispersed with an aqueous solution of 30 mM carboxyfluorescein dissolved in NaHCO_3_ (100mM) to generate a suspension of carboxyfluorescein entrapping MLV liposomes. The SUV liposomes are prepared by sonication as described above. Non-entrapped free carboxyfluorescein was eliminated by gel-filtration passing the SUV suspension through a 4B-sepharose column.

#### 2.2.4. Lipids Quantification

The quantification of lipid content in liposomes was measured by Stewart’s phospholipid colorimetric assay [21]. This method is based on the formation of a chloroform-soluble colored complex between phospholipids and ammonium ferrothiocyanate, which can be quantified by absorbance (485 nm). Briefly, an aliquot of 5 µL of the liposome suspension was dissolved in an assay tube containing 2 mL of chloroform and 2 mL of ammonium ferrothiocyanate solution. The ammonium ferrothiocyanate solution was made dissolving 27.03 g of ferric chloride hexahydrate (Sigma Aldrich Cat. no. F2877, Madrid, Spain) and 30.4 g of ammonium thiocyanate (Sigma Aldrich Cat. no. 221988, Madrid, Spain) in distilled water for a final volume of 1 L. Similarly, for the standard curve, a series of tubes were prepared using a phosphatidylcholine solution. The tubes were vigorously vortexed for 1 min to mix the organic and aqueous phases and allowed to stand for 10 min to phase separation. The complexes between the ferrothiocyanate ion and the phospholipids were present now in the organic phase, from which an aliquot was taken to measure the Absorbance at 488 nm in the colorimeter. The quantification of phospholipid content in the liposome samples was made by the interpolation of the optical density measures at 485 nm in the standard curve, prepared from a phosphatidylcholine solution (Sigma Aldrich Cat. no. P3556, Madrid, Spain) in chloroform at a concentration of 0.1 mg/mL.

#### 2.2.5. Preparation of Lipopolyplexes

Lipopolyplexes were made by mixing the MLV or SUV liposomes suspension with a previously prepared polyplex solution for spontaneous charge-charge lipopolyplex complex formation. The polyplex component must be incubated for 15 min before the liposome addition. The resulting lipopolyplex suspension was vortexed and incubated for at least 1 h at room temperature before utilization. For hydrosoluble protein antigens, lipopolyplexes were made with MLV-FTSM:CH:DP 5:4:1 liposomes loaded with TMP protein from B16 cells extract and polyplex suspension of 0.5 µg/µL DNA (p2F or p2F *m*GM-CSF plasmid) in 5% glucose. A volume of polyplex suspension containing 10 or 50 µg DNA was mixed with the appropriate number of TMP-loaded liposomes. Then, it was vortexed and incubated for 90 min prior to its administration. The correct dose of liposomes for each lipopolyplex was achieved by mixing appropriate amounts of two MLV-FT liposome suspensions loaded with 5% glucose solution or TMP proteins, respectively, to prepare lipopolyplexes with a dose of 20 µg TMP or 2 µg TMP. For lipid GD3-bearing lipopolyplexes, 25 micromoles of lipid mix solution in organic solvent (CH3Cl) were prepared using two different lipid formulations, SM:CH:DP:GD3 (4:4:1:1) and SM:CH:GD3 (5:4:1). The MLV vesicles were made with 500 µL of 5% glucose solution; and 200 µL of the MLV liposomes were subjected to four cycles of sonication to obtain a SUV liposome suspension, as described above. The lipid content was quantified by Stewart’s method, and the MLV and SUV liposome suspensions were adjusted to 0.01 µmol lipid/µL by adding 5% glucose solution. Lipopolyplexes were prepared by adding the liposomes on a polyplex solution containing 0.5 µg/µL of pMOK plasmid in 5% glucose and were incubated for one hour at room temperature before administration to mice.

#### 2.2.6. Electrophoretic Lipopolyplex Characterization

Lipopolyplex was prepared as indicated using SM:CH:DP (5:4:1) carboxyfluorescein-encapsulating SUV liposomes. Samples of 10 µL of liposomes or lipopolyplex suspension were treated with 6 µL of 1% Triton X-100, 6 µL of Tris-acetate buffer, 6 µL of 5% heparin solution, and 3 µL of 2% Triton X-100 plus 3 µL of 10% heparin. The samples were incubated for 15 min before loading in the agarose gel. The electrophoresis was conducted in 0.8% agarose gel with Tris-acetate buffer (40 mM, pH 8) at 60 mV for 40 min. The gel was stained with ethidium bromide before examination under fluorescence illumination.

#### 2.2.7. Vaccination Assays

Female 8–10 weeks C57BL/6 strain mice were divided into cages in groups of 5 individuals and housed in standard housing conditions. Immunization assays were approved by the Biological Research Committee of the University of Valencia (Spain). In the case of vaccinations with water-soluble protein (TMP) antigens, we used mice with induced melanoma that were divided into 8 groups of five individuals. On day 0, the tumor challenge was added by injecting 100,000 viable murine melanoma B16 cells in the right leg. The TMP vaccine (100 µL) was administered subcutaneously in the left leg with the corresponding doses of lipopolyplex or controls every 14 days (days −21, −7, and +7). In vaccinations with a lipid antigen (GD3), the mice without tumor challenge were distributed into 7 groups of five individuals. Mice were vaccinated on days 0, 20, and 40. A dose of 130 µL of lipopolyplex (0.3 µmol lipids/50µg DNA) or the corresponding controls were injected intradermically on the back of the animal. The lipopolyplex assayed contained 0.03 micromoles of GD3 lipid antigen located in the liposome lipid bilayer and 50 µg of pMok plasmid (containing the murine GM-CSF gene) in the polyplex component. In all cases, before the administration of each dose of vaccine, up to 200 µL blood sample was taken from the animal for subsequent experiments to characterize the immune response induced by the vaccine.

#### 2.2.8. Obtaining Plasma and Titration of Specific IgG Against TMP

ELISA plates were incubated with TMP protein extract from B16 cells (0.8 µg protein/mL in NaHCO3 buffer pH 9.6) and then treated with 1% BSA solution to neutralize the non-specific binding sites. Up to 200 µL blood samples were obtained from the tail vein of five mice in each vaccination group. These samples were centrifuged for 5 min at 3000 rpm to separate the plasma and then equivalent volumes from each animal were mixed to form a pool and then kept at −20 °C until its analysis. The pooled plasma sample was diluted with dilution buffer (1% BSA and 0.1% Tween 20 in PBS) before adding it to the ELISA plate. For the IgG and IgG1 titrations, a 1/1000 dilution was used; for IgG2a titrations, 1/100 dilution was required. As secondary antibody for total immunoglobulins, goat antisera to total IgG (Biocheck, Scarborough, USA) was used at 1/10,000 dilution. For specific subtypes, 1/1000 dilution mouse IgG subclasses (Sigma-Aldrich, Madrid, Spain) were used. The plates were developed by adding orthophenylenediamine and hydrogen peroxide and measuring the absorbance of the samples at 492 nm in duplicate.

#### 2.2.9. GD3 Antigen Serology Assays

Purified gangliosides were dissolved in ethanol (1 µg/mL) and adsorbed to 96-well ELISA plates (Immunolon II, Dynatech, New Jersey, USA) by organic solvent evaporation at room temperature. Plates were rehydrated with 200 µL of 1.5% skimmed milk in PBS and then 50 µL of diluted serum samples were added. After incubation for 2 h at 37 °C, plates were washed with washing solution (PBS 0.1% and Tween-20) (E. Merck, Darmstadt, Germany), and left for 60 min with 50 µL of alkaline phosphatase-labeled anti-mouse immunoglobulin anti-serum (Dako, Glostrup, Denmark) at 1:1000 dilution in washing solution. After incubation, the plate was washed (several wash cycles) and incubated for another 60 min at 37 °C with para-nitrophenyl phosphate (E. Merck, Darmstadt, Germany). The absorbance of the pre-immune sera was subtracted from that of the immune sera to give the corrected absorbance. To eliminate the effect of nonspecific antibodies, additional ELISA plates were prepared identically but without purified GD3 ganglioside, and the absorbance from this plate was subtracted from the absorbance of each of the serum plates. The serological titer in ELISA was defined as the highest dilution yielding a corrected absorbance of 0.100 or greater. The specificity of the responses against GD2, GD1a, GD1b, GM1, GM2, and GM3 was analyzed by ELISA. For isotype and IgG subclass characterization, we used 50 µL of affinity-purified alkaline phosphatase-labeled anti-mouse IgM, IgG1, IgG2a, IgG2b, and igG3 antisera (Zymed Laboratories Inc., San Francisco, CA, USA) appropriately diluted (1:1000) in washing solution instead of alkaline phosphatase-labeled anti-mouse immunoglobulin antiserum. These polyclonal antibodies were previously checked with mouse IgG subclasses IgA, IgM, and light chains, and no cross-reactivity was detected.

#### 2.2.10. Electron Microscopy

The evaluation of the size and morphology of the complexes was performed by transmission electron microscopy. Special grids for electron microscopy were treated with 0.4% Fomvar’s solution ethylene chloride and covered with a layer of graphite. Once the grid was prepared, a drop of sample (prepared following the usual protocol) was deposited, staying in contact for 5 min. After washing the sample with PBS, it was dried with absorbent paper, and a drop of 1% phosphotungstic acid was added on the grid, for two minutes. The grid was then dried with absorbent paper and stored until study in a Philips CM-10 100 KW electron microscope (Philips, Amsterdam, The Netherlands). Phosphotungstic acid can provide liposome negative stain

#### 2.2.11. Tumor Volume Measurement and Mice Survival Rate

The tumor was implanted intradermally in mice leg, causing an increase of paw size. Tumor growth in mice was also visually monitored and measured with a caliper in two dimensions: A (long diameter) and B (short diameter). Tumor volume was calculated with the formula: *V* = (*A* × *B*^2^)/2. Survival rate caused by tumor growth was registered.

#### 2.2.12. Expression of mGM-CSF in B16 Murine Melanoma Cells after Transfection with pMok-GMCSF or p2F-GMCSF

B16 murine cells at 80% confluence were transfected with 10 µg/mL dose of pMok-GMCSF or p2F-GMCSF plasmids employing PEI (polyethyleneimine, 25 kDa) as vector employing a PEI:DNA mass ratio of 1.41:1. After transfection, cells were cultured at 37 °C and 5% CO_2_ for 6 days. Culture medium was collected at days 1, 2, 3, and 6, centrifuged for 5 min at 3000 rpm to remove death cells and murine GM-CSF protein was determined with specific ELISA kit (BD OptEIA, RUO-555167, BD, NJ, USA) following the manufacturer protocol.

#### 2.2.13. Statistical Analyses

The results of IgG titration obtained in each group were compared with those obtained in all the other groups and statistical differences were determined by the two-way ANOVA (analysis of variance) method. Both statistical analyses and graphs were generated with Prism 5 (GraphPad Software, San Diego, CA, USA, 2011). We considered statistical differences when *p*-value ≤ 0.05.

## 3. Results

### 3.1. Lipopolyplex Preparation Method and Characterization

**Figure 2:**
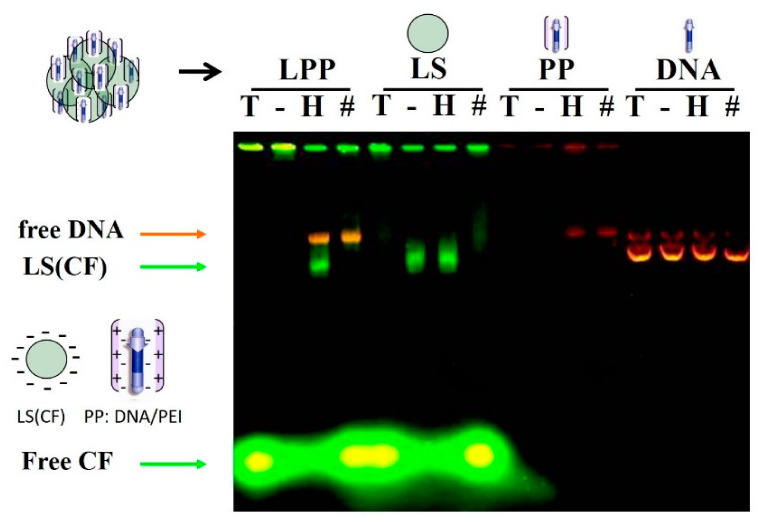
Characterization of the lipopolyplex by electrophoretic mobility shift assay (EMSA) assay. Triton X-100 (T), glucose 5% (-), heparin (H), or Triton X-100 plus heparin (#) were added to samples of lipopolyplex (lanes 1–4), liposomes (lanes 5–8), polyplex (lanes 9–12), and free plasmid p3C-EGFP (lanes 13–16) were added before loading on the gel. Electrophoresis ran at 80mV for 45 min before staining with ethidium bromide and visualizing it under UV trans-illumination. LPP: Lipopolyplex; LS: Liposome; PP: Polyplex; CF: Carboxyfluorescein.

The LPP was prepared in two consecutive stages. First, the plasmid was mixed with PEI to form the DNA/PEI polyplexes at an N:P (nitrogen:phosphate) charge ratio of 10:1. The PEI was added to the plasmid solution, and this order is essential for an adequate polyplex formation [22]. These polyplexes were incubated for 15 min before the addition of SUV liposomes. This mix was prepared in water or 5% glucose solution. The salts-containing media caused the formation of large aggregates after liposomes addition. The lipopolyplexes TEM images (Figure 1) show micron scale complexes composed by aggregation of multiple liposomes (negatively stained) surrounding more electro-dense areas where the polyplex (DNA-PEI) should be located. We performed an electrophoresis experiment to evaluate the interaction between the components that form the LPP, employing reagents, such as Triton X-100 and heparin, which affect the interaction between different components of the LPP (constituted with liposomes entrapping CF and DNA/PEI polyplex, respectively). The results were compared with those obtained with liposomes alone, polyplexes alone, and p3c-eGFP DNA plasmid alone (Figure 2). Samples were treated with a) Triton X-100 detergent (T), which solubilizes the liposomes, releasing the encapsulated CF; b) 5% glucose (G), as control; c) heparin (H), which binds to PEI and dissociates the polyplexes, releasing the plasmid; and d) Triton X-100 and heparin (T/H). Treatment with Triton X-100 dissolved the liposomes, causing the mid-size green bands to disappear and thickening the green fluorescence spot at the end of the gel that corresponds to the CF released from these liposomes. The control lipopolyplex sample treated with 5% glucose solution did not show any band except for the well, as it was not able to migrate through the gel pore, remaining retained. Treatment with heparin caused the appearance of mid-size green fluorescence bands, which we identify as free liposomes with CF as they were coincident in the electrophoretic migration with the control liposome bands. The orange band was identified as the plasmid liberated from the polyplex component. Combined treatment of lipopolyplexes with heparin and Triton X-100 produced only a plasmid DNA band, as the liposomes had been destroyed by the detergent. Samples were pre-incubated for 15 min with Triton X-100 and/or heparin before being loaded and run in the agarose gel.

### 3.2. Optimization of Lipopolyplex Preparation

**Figure 3:**
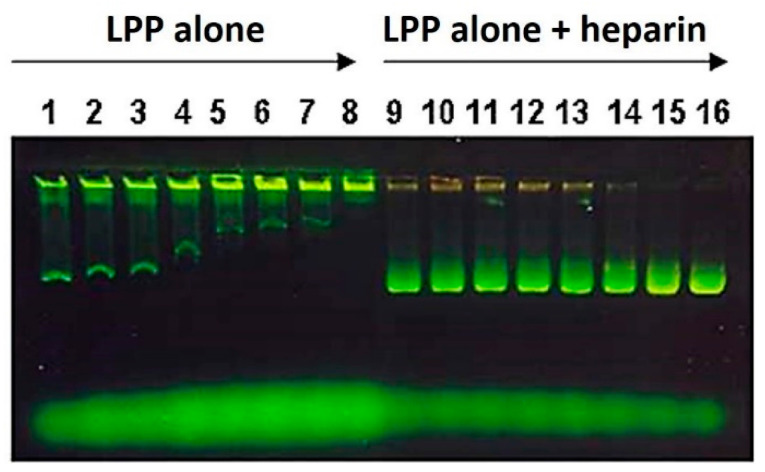
Determination of the optimal relationship between liposomes and polyplexes to form the lipopolyplex. The SUV SM: CH: DP lipopolyplex loaded with carboxyfluorescein was prepared at eight different mixing ratios by adding decreasing amounts of liposomes to a constant volume of the polyplex solution. On the left (lanes 1–8), samples of the lipopolyplex were loaded directly onto the gel. On the right (lanes 9–16), the samples of lipopolyplex were treated with heparin before loading on the gel.

To optimize the vector as a vaccine delivery system, it was necessary to define the optimal ratio between the two components (lipids and polyplexes) in order to prepare the lipopolyplex without excess of lipid component, avoiding the presence of free liposomes in the preparation. With this aim, we used a variant of the EMSA (electrophoretic mobility shift assay) test. LPP was prepared by adding decreasing volumes of a stock solution of anionic liposomes (SUV SM:CH:DP) to a constant volume of polyplex suspension (0.5 µg/µL of DNA in 5% Glucose), obtaining different lipid/DNA ratio lipopolyplex preparations: 0.048, 0.024, 0.021, 0.018, 0.015, 0.012, 0.009, and 0.006 mmol lipid/µg DNA. These lipopolyplexes were loaded in an agarose gel, alone or with heparin, which contributes to dissociating the two components of the particles, liposomes and polyplexes. Electrophoresis allowed defining the optimal liposomes/polyplexes ratio to form the lipopolyplex. The lipopolyplexes alone samples are located in the left part of the gel (lanes 1 to 8), whereas the corresponding heparin-treated lipopolyplexes samples are located on the right (lanes 9 to 16). As shown in Figure 3, lipopolyplexes without heparin treatment did not migrate through the agarose gel, remaining retained in the well, with the excess of liposomes appearing as green bands of free liposomes. These bands of free liposomes decreased progressively until it completely disappeared at the optimal ratio of 0.006 mmol lipid/µg DNA, which was chosen for the immunization assays with lipid antigens (0.3 micromoles of liposomes/50 µg of plasmid). In this assay, the free plasmid DNA bands are inversely proportional to the liposome/polyplex ratio. In the final lanes, with lower amounts of liposomes, heparin can dissociate the polyplex easily. It can be observed that there is not DNA within the well because it migrates through the gel until the same band of liposomes, thus producing the more yellowish color of the last lanes in the gel.

### 3.3. Vaccination With Hidrosoluble Proteins

The multicompartmental lipopolyplexes carried an extract of TMP from melanoma B16 murine cells and an expression plasmid containing the murine GM-CSF gene. The liposomes used (PC:CH:DP 5:4:1 or SM:CH:DP 5:4:1) were prepared by MLV-FT method. Mice received a dose of lipopolyplex containing 10 (GM10) or 50 (GM50) micrograms of mGM-CSF encoding plasmid (p2F or pMok) and 2 or 20 µg of tumor membrane proteins (TMP2 or TMP20). Groups of mice that received 5% glucose solution, liposomes loaded with 20 µg of TMP, and lipopolyplex with empty p2F plasmid (p2F50Ø/TMP20) were used as controls. Mice were injected with 100 µL in each vaccination in the right leg in accordance with the following vaccination groups of the Table 1.

**Figure 4:**
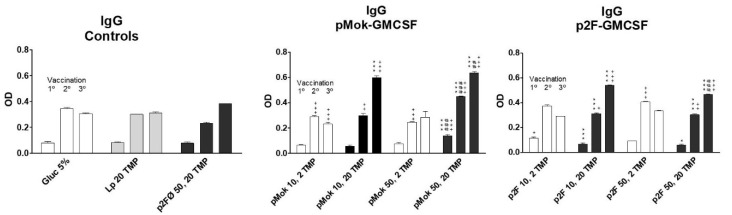
Total IgG response obtained after each vaccination dose in the different formulations of multicompartmental lipopolyplex loaded with a water-soluble protein extract (TMP). Lipolyplexes contained 10 or 50 µg of plasmid (pMok or p2F) bearing murine GM-CSF gene and 2 or 20 µg of TMP. * symbol is employed to define the statistically significant difference due to the amount, 2 or 20 µg, of TMP (* *p* < 0.05; *** *p* < 0.001) in each vaccination. # symbol indicates the statistical significance due to the amount, 10 or 50 µg, of GM-CSF (## *p* < 0.01; ### *p* < 0.001) in each vaccination. ^+^ symbol is employed to define the statistically significant difference with respect to Glucose 5% control group. (^+^ *p* < 0.05; ^+ +^ *p* < 0.01; ^+ + +^ *p* < 0.001) in each vaccination.

**Figure 5:**
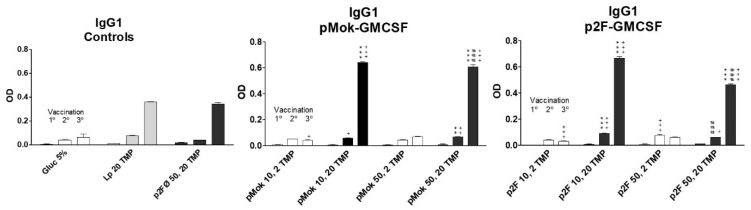
IgG1 response obtained after each vaccination dose in the different formulations of multicompartmental lipopolyplex loaded with a water-soluble protein extract (TMP). Lipolyplexes contained 10 or 50 µg of plasmid (pMok or p2F) bearing murine GM-CSF gene and 2 or 20 µg of TMP. * symbol is employed to define the statistically significant difference due to the amount, 2 or 20 µg, of TMP (** *p* < 0.01; *** *p* < 0.001) in each vaccination. # symbol indicates the statistical significance due to the amount, 10 or 50 µg, of GM-CSF (### *p* < 0.001) in each vaccination. ^+^ symbol is employed to define the statistically significant difference with respect to Glucose 5% control group. (^+^ *p* < 0.05; ^+ +^ *p* < 0.01; ^+ + +^ *p* < 0.001) in each vaccination.

**Figure 6:**
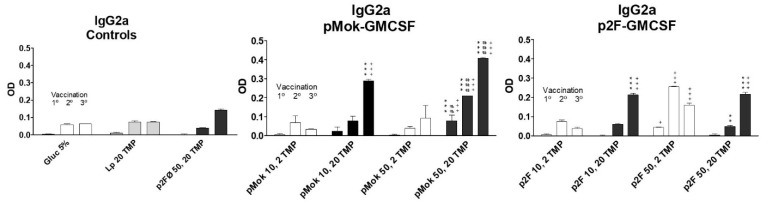
IgG2a response obtained after each vaccination dose in the different formulations of multicompartmental lipopolyplex loaded with a water-soluble protein extract (TMP). Lipolyplexes contained 10 or 50 µg of plasmid (pMok or p2F) bearing murine GM-CSF gene and 2 or 20 µg of TMP. * symbol is employed to define the statistically significant difference due to the amount, 2 or 20 µg, of TMP (** *p* < 0.01; *** *p* < 0.001) in each vaccination. # symbol indicates the statistical significance due to the amount, 10 or 50 µg, of GM-CSF (## *p* < 0.01; ### *p* < 0.001) in each vaccination. ^+^ symbol is employed to define the statistically significant difference with respect to Glucose 5% control group. (^+^ *p* < 0.05; ^+ + +^ *p* < 0.001) in each vaccination.

In all cases, mice were vaccinated (right leg) with a single dose per week, in weeks –3, −1, and +1 (days –21, −7, and +7), with respect to tumor injection (day 0) of 100,000 wild type B16 cells in the left leg. Blood samples were taken on day −22 with respect to the tumor challenge (day 0) and before each vaccination with lipopolyplexes (days −21, −7, and +7, respectively). The titer of anti-TMP IgG and specific IgG1 and IgG2a immunoglobulins generated after each vaccination were measured by ELISA in duplicate, and the titers obtained are represented in Figure 4, Figure 5 and Figure 6.

High doses of TMP (20 µg) achieved significantly (*p* < 0.001) higher IgG immunoglobulin titer than low doses of antigen (2 µg) from the third vaccination dose (Figure 4) when administered with GM-CSF. We consider that the expression of GM-CSF prepares the immune system to respond to the stimulus (TMP) but to trigger the response, an amount higher than 2 µg is required to activate the antibodies production. The administration of liposomes loaded with 20 µg of TMP alone did no trigger the increase of immunoglobulins levels. We consider the elevation of Ig after 2° and 3° vaccinations in glucose 5% and liposomes with TMP groups could be due to the immune response against the tumor. Comparing the results of lipopolyplexes constituted with 20 µg of TMP, the immunoglobulin titer was significantly higher (*p* < 0.001 in 1° and 2° vaccination and *p* < 0.05 in 3° vaccination) with lipopolyplexes containing 50 µg of plasmid than with lipopolyplexes containing 10 µg plasmid dose in pMok groups (Figure 4, central panel). In contrast, in p2F plasmid groups (Figure 4, right panel), the low dose of GM-CSF mediated significantly (*p* < 0.001 in 3° vaccination) higher titer of total IgG than 50 µg dose. On average, pMok plasmid induced slightly higher levels of total IgG than p2F.

The increase in humoral titer after the third vaccination for high protein doses was even more pronounced (*p* < 0.001) in IgG1 immunoglobulins (Figure 5). The groups vaccinated with high doses (50 µg) of GMCSF did not generate higher titers of IgG1 but lower, in contrast to what occurred with total IgG. IgG2a levels (Figure 6) increased with successive vaccination, with the highest titers obtained after the third vaccination, especially for the groups vaccinated with high doses of TMP. However, in low-dose protein lipopolyplexes, there was a greater increase of IgG2a after the second vaccination, the group vaccinated with p2F-GMCSF (50 µg)/TMP (2 µg) obtaining the highest titer of anti-TMP IgG2a antibodies after the second dose of vaccine (day +15), significantly higher (*p* < 0.05) than that obtained with p2F-GMCSF (50 µg)/TMP (20 µg). In pMok groups, 50 µg of GMCSF mediated higher concentrations of IgG2a than 10 µg (*p* < 0.05 after 1° vaccination; and *p* < 0.001 after 2° and 3° treatments), resulting in higher levels than those observed with p2F plasmid in all cases. In p2F groups, DNA doses did not have effect on the final titer of IgG2a.

Although the results were not statistically significant, groups treated with vaccines of low GM-CSF gene dose and 20 µg TMP (p2F 10/TMP 20 or pMok 10/TMP 20) showed delayed tumor growth (Figure S1). Plasmid p2F also mediated longer survival rate (Figure S2), especially when lower GM-CSF dose was employed. These results, added to the fact that p2F plasmid mediates lower production of GM-CSF (Figure S3), corroborates that supramaximal doses of GM-CSF limit its pharmacological effect, reducing the antitumor immune response, as previously reported [14,15]. The other treatments did not prove different results to those obtained with controls.

### 3.4. Vaccination with Lipidic Antigen GD3

To test the capacity of the multicompartmental lipopolyplex as immunization vector with lipid antigens and the specificity of the response against a single antigen, we prepared lipopolyplexes with GD3 lipid antigen and murine GM-CSF gene plasmid. Mice were vaccinated on days 0, 20, and 40 with an intradermic injection on the back with control solution or 130 µL dose of the lipopolyplex vaccine. The vaccination dose was 0.03 µmoles of GD3/50 µg of DNA. Animals were divided into seven vaccination groups of five mice that received three successive doses every 20 days. Vaccination groups were as indicated in Table 2.

A control group with SUV liposomes alone was not included in the assay, since this type of liposome had already shown not to induce humoral response in previous work in our laboratory [23]. Blood samples were collected on days 0, +20, +40, and +60, before each vaccine administration. Serum was analyzed by ELISA to determine the levels of total IgM and IgG immunoglobulins (Table 3), expressed as the maximum dilution with Ig detection (absorbance ≥0.100). Results showed a progressive increase in immunoglobulins levels after each dose of vaccine, being more pronounced in liposomes or lipopolyplexes formulated with DP (dicetylphosphate). Lipopolyplexes (mGM-CSF DNA and GD3 lipid antigen) formulated with SUV liposomes with DP achieved the highest titer (1:800) of IgG immunoglobulins, higher than that generated by the corresponding control group vaccinated with liposomes alone.

The following serological tests were performed using the sera obtained after the third vaccination dose. In specific immunoglobulin subtypes’ titration (Table 4), the production of IgG2a immunoglobulins stands out. Once again, the lipopolyplex made with SUV SM:CH:DP liposomes achieved higher IgG2a titer than the rest of the lipopolyplexes or liposomes tested in the trial. The titers of IgG1 (1:25) and IgG2b (1:25) subtypes were similarly low in all groups. IgG3 titers were slightly higher but similar with two lipopolyplexes and the control MLV liposomes formulated with DP. Regarding the specificity of the anti-GD3 response, a cross-reactivity test of the sera against other antigens similar to GD3 (GM1, GM2, GM3, GD1b, and GD2) was carried out and a very specific anti-GD3 response was observed (Table 5), with only very low reactivity against Gd1b ganglioside.

## 4. Discussion

Gene therapy is a promising approach for the treatment or prevention of acquired and genetic diseases. One of the main limitation of these therapies consist in the limited delivery of the nucleic acids due to their size. For this reason, different types of vectors or carriers have been developed. Viral vectors have been widely studied and, although they offer efficient delivery of nucleic acid product, they present some safety issues. Some of these problems can be circumvented by employing non-viral vehicles such as cationic liposomes or polymers, which form complexes with DNA to favor its transport to the target. These vehicles have been developed and evolved to improve their efficacy of transport and delivery [24,25] and can be combined to synergize their capacities, forming lipopolyplexes. The multicompartmental lipopolyplexes permit the incorporation of antigens with diverse chemical nature. Hydrophobic molecules, such as lipids, can be placed in the lipid bilayer during the formation of liposomes, simply by adding them to the initial lipid mixture. Hydrophilic molecules, such as proteins, can be entrapped in the aqueous liposome compartment. Protein antigens could be also encoded in the plasmid of the polyplex component [26]. Unlike other systems, multicompartmental lipopolyplexes can simultaneously carry water soluble and hydrophobic antigens associated with plasmids encoding adjuvants, such as immunostimulatory cytokines, chemotactic factors, or other protein antigens. Thus, one advantage of lipopolyplexes is their versatility in antigen transport, offering very wide possibilities. On the other hand, the multicompartmental lipopolyplexes present high capacity to transport these genes and antigens inside their components. The complex formation has been demonstrated by a fluorescent-liposomes EMSA assay with the selective dissociation of lipopolyplexes into their components and by TEM. Treatment with Triton X-100 solubilizes the liposomes, releasing the encapsulated carboxyfluorescein, while the addition of heparin releases the liposomes from the complex and separates the plasmid from the PEI. The green fluorescent areas observed at the lower part of gel lanes are free carboxyfluorescein. It is more visible in the samples treated with Triton X-100, which breaks down liposomes, releasing CF. The pore size of agarose gels is in a range between 10-100 nm [27], which is consistent with the mobility through the gel of SUV liposomes, whose diameters size are in ranges of 20–80 nm [28].

One of the most remarkable features of the multicompartmental lipopolyplex is that both antigens and plasmids are simultaneously introduced to the same cell that incorporates the lipopolyplex particle. Furthermore, it occurs in controlled quantities defined by the stoichiometry of the components used in the particle preparation. The ratio of loaded antigen with respect to immunostimulating cytokine (encoded in the plasmid of DNA) can be easily controlled, and the cells will receive these two components in a predefined proportion. However, the saturating amount of liposomes required to form lipopolyplexes with polyplexes needs to be established. Otherwise, the presence of free liposomes that have not been complexed with polyplexes would represent heterogeneous vaccination preparations and immune response may not be correlated only to the lipopolyplex. It is essential to have a characterization method since it is necessary to precisely correlate the antigen doses in the lipopolyplex form with the response obtained. Some lipopolyplex characterization methods are available in the literature [29], but they are complex, sometimes requiring specialized instruments such as HPLC. A variant of the fluorescent EMSA assay, in which increasing amounts of liposomes were added to a fixed amount of polyplex to form the lipopolyplexes, was designed to determine the optimal ratio between these two components. Lipopolyplexes were made by adding different quantities of liposomes to the polyplex component. They were then dissociated by mixing with heparin, and immediately loaded onto the gel without incubation time. Under these conditions, heparin undoes the liposome-polyplex complexes. Polyplexes dissociation was inversely proportional to the liposomes amount in the mix, with free plasmid bands being observed to b mixed with liposomes in the complexes formed with lower liposomes/polyplex ratios. This observation suggests that the anionic charge of heparin easily displaces the liposome-polyplex union but the employed concentrations result less efficient to dissociate the DNA/PEI complex, due to the abundant anion charge of DNA. Lipopolyplexes made with an excess of liposomes showed a band of free unbound liposomes, which did not appear in the optimal ratio formed lipopolyplex. In this way, we can obtain preparations of lipopolyplexes without free liposomes, allowing the entire dose of antigens to be in the form of lipopolyplex.

The multicompartmental lipopolyplex provides very interesting characteristics as a vaccination vector. Liposomes are prepared independently so that they can be made by any of the well-established methods to load them with proteins or other molecules [28]. The efficiency of the liposomal encapsulation may be quantified before using them to constitute the lipopolyplex. The antigen dose can be adjusted by mixing the protein-loaded liposomes with void liposomes before lipopolyplex preparation, so defining the antigen doses used. The quantities of molecules that a single lipopolyplex particle delivers are high, as multiple units of liposomes and polyplexes are associate with each other, providing a high antigen and gene load to the cell that phagocyte the particle. The multiple liposomes provide the complex with higher stability against serum [30,31]. With respect to the polyplex component, unlike others lipopolyplexes that situate them in the core of the particle, in the multicompartimental lipopolyplexes, they are externally associated with the liposomes, which would favor the proton sponge effect and the rupture of the endosome. The progressive acidification of the endosome is interfered by the primary and secondary amino groups of the PEI polymer, which can sequester and immobilize a large number of protons within the endocytosis vesicle. As a result, chloride ions are pumped into the vesicle, to maintain the neutrality of electrical charges, strongly increasing the entry of water by osmotic effect, which finally causes the rupture of the endosomal membrane [32]. The outer location of the polyplexes makes it easily accessible to the internal environment of the endosome, favoring the proton sponge effect that allows access to the cell cytosol.

The wide versatility to deliver molecules of diverse chemical nature is another advantage of the vehicle design. Antigens of a very varied chemical nature can be included since it accepts both hydrosoluble and hydrophobic molecules, and even protein antigens encoded within the plasmid of the polyplex component. Another interesting possibility is the delivery of mRNA. Some recent assays have shown strong antitumoral immunostimulation in vaccine assays using lipopolyplexes loaded with antigen-encoding mRNA alone or in combination with a lipid adjuvant included in the lipid bilayer [33,34]. The multicompartmental lipopolyplex may load mRNA incorporated in the form of RNA-loading liposomes or as PEI:RNA polyplexes [35]. However, the bioavailability and efficacy of mRNA after its delivery should be previously confirmed. In the present work, we took advantage of this feature to include in the formulation the murine GM-CSF gene DNA aiming to induce the production of this cytokine to stimulate the immune response against the employed antigen. The stoichiometric relationships between the components of the particle are easy to control during its preparation, using the EMSA assay to define an optimal liposome/polyplex ratio for lipopolyplexes preparation. The molecule-loading capacity of these carriers can be exploited to provide additional characteristics. For example, heparin is able to dissociate the lipopolyplexes and liberate the plasmid from the polyplex component [10]. We speculate that the heparin encapsulation in the liposomes may favor, at least partially, the dissociation of DNA from the lipopolyplex whether heparin reaches efficacious concentration in the endocytosis vesicle. This could favor the release of naked DNA in the cytoplasm when the endosome is destabilized by the proton sponge mechanism [30,36,37]. In addition, the delivery of naked DNA to cytoplasm after lipopolyplex dissociation increases its opportunity to access the nuclear compartment.

Antitumor immunization assays can be performed with a single antigen or using multiple antigens. The single antigen model can confirm antigen-specific immune response. In contrast, in multiple antigen vaccination, the change in immunoglobulin titer can only be measured in response to immunization. We have tested the multicompartmental lipopolyplex loaded with chemically different antigens, in a multiple antigen model with hydrosoluble proteins (TMP) and in a single antigen model with a lipid antigen (GD3). In both cases, multi-compartmental lipopolyplexes have been shown capable of inducing an immunoglobulin response. For testing the immunization with TMP protein antigens, we used MLV-FT liposomes to form the lipopolyplex, since they have higher protein encapsulation yield that other types [28].Tumor cells are heterogeneous and, although vaccine with TMP is less selective, it offers advantages regarding the number of different tumor targets included in this antigenic mix. It then reduces the risk of clonal selection and the vaccine efficacy loss.

The multicompartmental lipopolyplex achieved a higher humoral immune response than in mice vaccinated with free TMP proteins or encapsulated in liposomes. Among the various lipopolyplexes used, the one formed with the highest dose of antigen (20 µg of TMP) and the lowest dose of cytokine (10 µg of GM-CSF DNA) produced the highest IgG titers after the third vaccination dose, a trend that was also maintained in the specific immunoglobulin subtypes IgG1 and IgG2a. The result that lower dose of plasmid is more efficient is not surprising. In this respect, our results show that the highest survival rate was achieved with lower doses of p2F-GMCSF, which is the plasmid with lower production of the cytokine. These results are in agreement with previous observations that supramaximal doses of GM-CSF (as it occurs with other drugs) limit its pharmacological effect, adversely affecting to the antitumor immune response [14,15,38].

Regarding the specific isotypes, IgG1 increases very significantly after the third immunization administration with two high-antigen dose (20µg TMP) lipopolyplexes, especially in GM10/TMP20, which again gives the highest titer. This trend is maintained for IgG2a immunoglobulin. However, it is observed that low-protein (2µg TMP) lipopolyplexes mediate higher IgG1 titer after the second vaccine dose than after the third, especially with GM50/TMP2, which yields the highest IgG2a titer. Concluding, better immune responses were induced by loading lipopolyplexes with the higher dose of proteins but moderate doses of the plasmid encoding GM-CSF cytokine, since higher DNA doses could even reduce the humoral response obtained.

For the assay with lipid antigens, we have chosen the ganglioside GD3, an abundant lipid in membranes of melanoma cells with the aim of confirming both the efficacy and specificity of the immune response. GD3 was incorporated into the lipid bilayer of the MLV or SUV liposomes. Lipopolyplexes showed superior efficacy compared to liposomes in developing humoral immune responses. In particular, the lipopolyplex made up of SUV liposomes demonstrated greater efficiency in the production of antibodies and induction of immunoglobulins-mediated cytotoxicity, with the response also being specific. The specific IgG1, IgG2a, and IgG3 subtypes that appear after the immunoglobulin switch produced in vaccination were studied to better characterize the humoral response obtained. In particular, the LPP made up of SUV liposomes achieved higher of IgG2a titer than the rest of lipopolyplexes, suggesting the appearance of a possible response and cellular memory, of maximum interest in antitumor immunization [39]. Multicompartmental lipopolyplex-based delivery system incorporating hydrophilic and lipophilic may provide a promising vaccination tool for the diseases requiring humoral immunity.

Although we did not observe any toxic effects that could be attributed to LPP vaccine treatment, further dose-response toxicity studies will be required prior to its clinical translation.

## 5. Conclusions

The multicompartmental lipopolyplex associates multiple polyplexes and liposomes in the same particle that can jointly carry hydrophilic and/or hydrophobic antigens plus DNA of interest (encoding antigens or cytokines) to the same cell.

We have developed a simple and affordable electrophoretic assay that demonstrates the formation of the complex and defines the precise ratio that permits preparing a lipopolyplex vaccination product without free liposomes. Efficacy and response specificity of this vehicle was demonstrated in a murine melanoma model. Lipopolyplex proved its efficacy employing TMP as spread spectrum hydrophilic antigens. However, due to the diversity of antigens in the protein extract, it was not possible to verify the specificity of the response obtained. The vaccination assay with a single lipid antigen (GD3) allowed us to verify its specificity.

Our multicompartmental lipopolyplex allows not only to administer compounds of different nature, but also to control in a more specific way the release (with heparin, for example) of each element to manage the times and the place where the different components are delivered.

## Figures and Tables

**Table 1:** Immunization assay groups with the multicompartmental lipopolyplex loaded with hydrosoluble protein antigens (TMP).

Vaccine Group	Type	Lipid Composition	Plasmid	GM-CSF (µg)	TMP (µg)
5% glucose	-		-	-	-
TMP20	liposome	PC:CH:DP	-	-	20
p2F50/TMP20	lipopolyplex	PC:CH:DP	p2F	-	20
p2FGM10/TMP2	lipopolyplex	SM:CH:DP	p2F	10	2
p2FGM10/TMP20	lipopolyplex	SM:CH:DP	p2F	10	20
p2FGM50/TMP2	lipopolyplex	SM:CH:DP	p2F	50	2
p2FGM50/TMP20	lipopolyplex	SM:CH:DP	p2F	50	20
pMokGM10/TMP2	lipopolyplex	PC:CH:DP	pMOK	10	2
pMokGM10/TMP20	lipopolyplex	PC:CH:DP	pMOK	10	20
pMokGM50/TMP2	lipopolyplex	PC:CH:DP	pMOK	50	2
pMokGM50/TMP20	lipopolyplex	PC:CH:DP	pMOK	50	20

**Table 2:** Immunization assay groups with the multicompartmental lipopolyplex loaded with lipidic antigens (GD3).

Vaccine Group	Type	Lipidic Vesicle	Plasmid	GM-CSF (µg)	GD3 (µmol)
5% glucose solution	-	-	-	-	-
MLV(SM:CH:GD3)	liposome	Multilamellar	-	-	0.03
MLV(SM:CH:DP:GD3)	liposome	Multilamellar	-	-	0.03
MLV(SM:CH:GD3)/pMok	lipopolyplex	Multilamellar	pMok	50	0.03
SUV(SM:CH:GD3)/pMok	lipopolyplex	Small unilamellar	pMok	50	0.03
MLV(SM:CH:DP:GD3)/pMok	lipopolyplex	Multilamellar	pMok	50	0.03
SUV(SM:CH:DP:GD3)/pMok	lipopolyplex	Small unilamellar	pMok	50	0.03

**Table 3:** Serological responses to immunization with GD3 antigen after the first, second, or third dose of vaccine. Animals were immunized with liposomes bearing the GD3 antigen (SM: CH: GD3 or SM: CH: DP: GD3) or with lipopolyplexes bearing GD3 and the pMOK plasmid bearing GM-CSF cytokine gene. Titers of anti-GD3 immunoglobulins were determined by ELISA. The titers correspond with the maximal dilution of mice serum that yielded an optical density above 0.100. These determinations were performed in triplicate and only results with dispersion lower than 5% were accepted.

		TREATMENT GROUP						
		CONTROL	LIPOSOMES		LIPOPOLYPLEX			
			SM:CH:GD3	SM:CH:DP:GD3	SM:CH:GD3/pMok		SM:CH:DPGD3/pMok	
VACCINATION	Ig Type	5% Glucose	MLV	MLV	MLV	SUV	MLV	SUV
1° VACCINATION	IgM	0	1:25	1:50	1:25	1:25	0	1:100
	IgG	0	0	1:100	1:50	1:100	1:50	1:100
2° VACCINATION	IgM	0	1:50	1:200	1:25	1:25	1:50	1:200
	IgG	0	1:50	1:200	1:100	1:100	1:200	1:200
3° VACCINATION	IgM	0	1:50	1:100	1:100	1:50	1:50	1:100
	IgG	0	1:100	1:200	1:100	1:200	1:200	1:800

**Table 4:** Characterization of the specific isotypes of anti-GD3 immunoglobulins obtained after the third vaccination dose. Immunoglobulin titration was determined by ELISA. The titers correspond with the maximal dilution of mice serum that yielded an optical density above 0.100. These determinations were performed in triplicate and only results with dispersion lower than 5% were accepted.

	TREATMENT GROUP						
	CONTROL	LIPOSOMES		LIPOPOLYPLEX			
		SM:CH:GD3	SM:CH:DP:GD3	SM:CH:GD3/pMok		SM:CH:DPGD3/pMok	
Ig Type	5% Glucose	MLV	MLV	MLV	SUV	MLV	SUV
IgM	0	1:50	1:100	1:100	1:50	1:50	1:100
Total IgG	0	1:100	1:200	1:100	1:200	1:200	1:800
IgG1	0	1:25	1:25	1:25	1:25	1:25	1:25
IgG2a	0	1:25	1:25	1:100	1:100	1:200	1:400
IgG2b	1:25	1:25	1:25	1:25	1:25	1:25	1:25
IgG3	0	1:100	1:200	1:50	1:50	1:200	1:200

**Table 5:** Specificity of the anti-GD3 immune response in mouse plasma after the third dose of vaccine. Cross-reactivity against other gangliosides with a chemical structure similar to GD3 (GM1, GM2, GM3, GD1b, and GD2) was measured by ELISA in the samples obtained after the third vaccination dose. Plasma samples only exhibited slight reactivity against the GD1b antigen, while the cross-reactivity was negligible for the rest of the antigens. The titers correspond with the maximal dilution of mice serum that yielded an optical density above 0.100. These determinations were performed in triplicate and only results with dispersion lower than 5% were accepted. The symbol ± was employed to indicate that although the optical density average result of three replicates was below 0.100, the data of one out of three replicates resulted slightly higher than 0.100.

	TREATMENT GROUP						
	CONTROL	LIPOSOMES		LIPOPOLYPLEX			
		SM:CH:GD3	SM:CH:DP:GD3	SM:CH:GD3/pMok		SM:CH:DPGD3/pMok	
Total IgG	5% Glucose	MLV	MLV	MLV	SUV	MLV	SUV
GD3	0	1:100	1:200	1:100	1:200	1:200	1:800
GM1	-	-	-	-	-	-	-
GM2	-	-	-	-	-	-	-
GM3	-	-	-	-	-	-	-
GD1b	±	±	±	±	±	±	±
GD2	-	-	-	-	-	-	-

## Data Availability

Data is contained within the article or supplementary material.

## Supplementary Materials

The following are available online at https://www.mdpi.com/1999-4923/13/2/281/s1, Figure S1: Tumor volume; Figure S2. Survival rate; Figure S3. Expression of GM-CSF in B16 murine melanoma cells transfected with pMok or p2F plasmids bearing mGM-CSF gene
