# Supplementary material for: Multicompartmental Lipopolyplex as Vehicle for Antigens and Genes Delivery in Vaccine Formulations
Source: Pharmaceutics. 2021 Feb 19;13(2):281. doi: 10.3390/pharmaceutics13020281 (PMC7922173; doi:10.3390/pharmaceutics13020281)
Supplement: Supplementary file 1 [file pharmaceutics-13-00281-s001.pdf]

pharmaceutics

MDPI

# Supplementary Materials: Multicompartmental Lipopolyplex as Vehicle for Antigens and Genes Delivery in Vaccine Formulations

Isaías Sanmartín, Luis Sendra, Inés Moret, María José Herrero and Salvador F. Aliño *

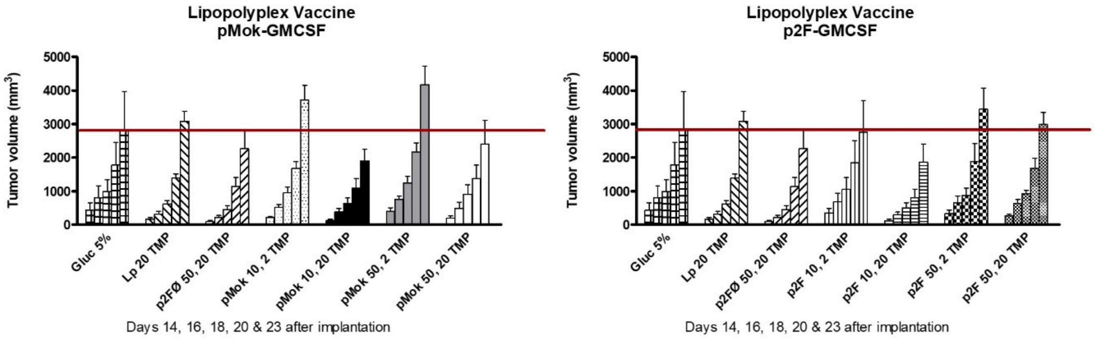


**Figure S1.** Tumor volume. Tumor cells were injected in mice leg on day 0. The paw volume of each mouse was measured on days 14, 16, 18, 20, and 23 after tumor implantation and the average value of each group was represented. Red line indicates the volume of control group (Gluc 5%). No statistically significant difference was observed among the different groups. Animals were vaccinated on days −21, −7, and +7 respect to tumor challenge. Gluc 5%: Mice treated with 5% glucose solution; LP 20 TMP: Mice treated with liposomes loaded with 20 µg TMP; p2FØ 50, 20 TMP: Mice treated with lipopolyplex containing 50 µg empty p2F plasmid and 20 µg TMP. pMok 10, 2 TMP: Mice treated with 10 µg pMok plasmid bearing GM-CSF gene and 2 µg TMP; pMok 10, 20 TMP: Mice treated with 10 µg pMok plasmid bearing GM-CSF gene and 20 µg TMP; pMok 50, 2 TMP: Mice treated with 50 µg pMok plasmid bearing GM-CSF gene and 2 µg TMP;pMok 50, 20 TMP: Mice treated with 50 µg pMok plasmid bearing GM-CSF gene and 2 µg TMP. p2F 10, 2 TMP: Mice treated with 10 µg p2F plasmid bearing GM-CSF gene and 2 µg TMP; p2F 10, 20 TMP: Mice treated with 10 µg p2F plasmid bearing GM-CSF gene and 20 µg TMP; p2F 50, 2 TMP: Mice treated with 50 µg p2F plasmid bearing GM-CSF gene and 2 µg TMP; p2F 50, 20 TMP: Mice treated with 50 µg p2F plasmid bearing GM-CSF gene and 2 µg TMP.

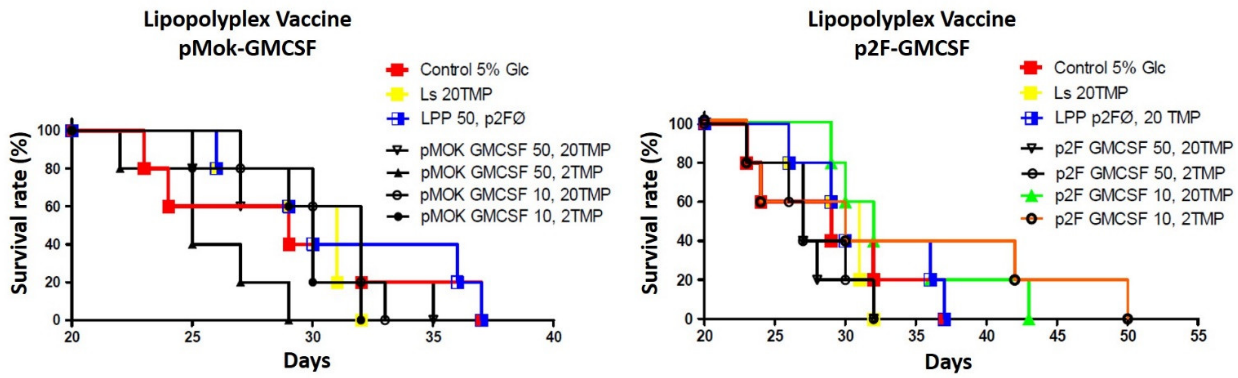


**Figure S2.** Survival rate. Kaplan Meier graphs of mice survival rates. The animals from control groups died on day 37 or before respect to tumor implantation. Vaccinations with pMok-GMCSF plasmid did not improve the survival rate. Vaccine formulations containing p2F-GMCSF slightly prolonged the survival (up to day 50), however with no statistically significant difference being observed. Animals were vaccinated on days −21, −7, and +7 respect to tumor challenge. Gluc 5%: Mice treated with 5% glucose solution; LP 20 TMP: Mice treated with liposomes loaded with 20 µg TMP; p2FØ 50, 20 TMP: Mice treated with lipopolyplex containing 50 µg empty p2F plasmid and 20 µg TMP. pMok 10, 2 TMP: Mice treated with 10 µg pMok plasmid bearing GM-CSF gene and 2 µg TMP; pMok 10, 20 TMP: Mice treated with 10 µg pMok plasmid bearing GM-CSF gene and 20 µg TMP; pMok 50, 2 TMP: Mice treated with 50 µg pMok plasmid bearing GM-CSF gene and 2 µg TMP; pMok 50, 20 TMP: Mice treated with 50 µg pMok plasmid bearing GM-CSF gene and 2 µg TMP. p2F 10, 2 TMP: Mice treated with 10 µg p2F plasmid bearing GM-CSF gene and 2 µg TMP; p2F 10, 20 TMP: Mice treated with 10 µg p2F plasmid bearing GM-CSF gene and 20 µg TMP; p2F 50, 2 TMP: Mice treated with 50 µg p2F plasmid bearing GM-CSF gene and 2 µg TMP; p2F 50, 20 TMP: Mice treated with 50 µg p2F plasmid bearing GM-CSF gene and 2 µg TMP.

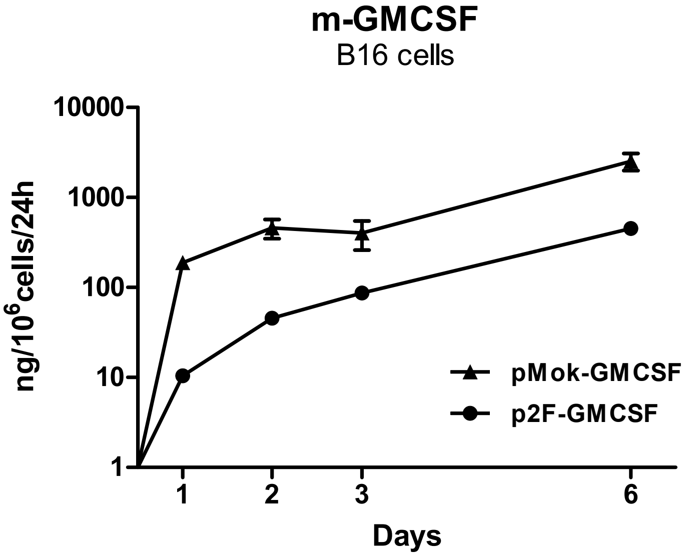


**Figure S3.** Expression of GM-CSF in B16 murine melanoma cells transfected with pMok or p2F plasmids bearing mGM-CSF gene. Cells were transfected with 10 µg/mL dose of plasmid and the presence of protein in culture medium was evaluated at days 1, 2, 3, and 6 of culture by ELISA.
